# A Developmental Perspective on the Origins of Morality in Infancy and Early Childhood

**DOI:** 10.3389/fpsyg.2018.01736

**Published:** 2018-09-20

**Authors:** Audun Dahl, Melanie Killen

**Affiliations:** ^1^Department of Psychology, University of California, Santa Cruz, Santa Cruz, CA, United States; ^2^University of Maryland, College Park, College Park, MD, United States

**Keywords:** morality, infancy, constructivism, social development, helping behavior, intergroup attitudes

## Abstract

Key constituents of morality emerge during the first 4 years of life. Recent research with infants and toddlers holds a promise to explain the origins of human morality. This article takes a constructivist approach to the acquisition of morality, and makes three main proposals. First, research on moral development needs an explicit definition of morality. Definitions are crucial for scholarly communication and for settling empirical questions. Second, researchers would benefit from eschewing the dichotomy between innate and learned explanations of morality. Based on work on developmental biology, we propose that all developmental transitions involve both genetic and environmental factors. Third, attention is needed to developmental changes, alongside continuities, in the development of morality from infancy through childhood. Although infants and toddlers show behaviors that resemble the morally relevant behaviors of older children and adults, they do not judge acts as morally right or wrong until later in childhood. We illustrate these points by discussing the development of two phenomena central to morality: Orientations toward helping others and developing concepts of social equality. We assert that a constructivist approach will help to bridge research on infants and toddlers with research on moral developmental later in childhood and into adulthood.

## Introduction

Key constituents of morality emerge early in ontogeny: by their fourth birthday, most children express obligatory judgments based on moral concerns with others’ welfare, rights, and fairness through spontaneous reactions and reasoning about perceived violations (Schmidt et al., 2012; Smetana et al., 2012; Dahl and Kim, 2014; Rizzo et al., 2016; for a review, see Killen and Smetana, 2015). How do newborns–seemingly unconcerned with moral issues–develop into preschoolers with moral capabilities that, in some ways, resemble those of adults?

Recent research on social cognitive abilities among infants and toddlers promises to shed light on how preschoolers come to reason about and judge moral issues. Most of the foundational work on cognitive developmental approaches to moral development focused on older children and adults (Piaget, 1932; Kohlberg, 1963, 1971; Turiel, 1983). In the last two decades, numerous researchers from social and moral developmental psychology (Killen and Smetana, 2015), as well as other areas in developmental psychology, have explored the presence of morally relevant concepts and behaviors in infants and toddlers (Bloom, 2013; Hamlin, 2013; Sommerville, 2015; Tomasello, 2016). Discussions about the origins of morality in infancy have often centered on whether some parts of morality are innate, or otherwise emerge independently of relevant experiences (Hamlin, 2013; Wynn and Bloom, 2014; Warneken, 2016). In these debates, key terms like “morality” and “innate” are often left undefined (Dahl, 2014).

In this article, we argue that explaining major transformations in early moral development requires a new lens, one that bridges the gap between infancy and childhood. This article makes three proposals for how to integrate research on very young children with research on moral development in later childhood. First, we propose that research on moral development needs explicit definitions of morality and other central concepts. Second, developmental acquisitions involve both genetic and environmental factors, and research on moral development would benefit from eschewing the dichotomy between innate and learned characteristics. Third, there are fundamental differences between the capabilities of infants and toddlers and the moral capabilities of older children. Within our framework, infants and toddlers demonstrate important precursors to morality, but lack core components of a developed morality. In elaborating on this third claim, we discuss age-related changes regarding young children’s orientations toward helpful behaviors and toward generalizing moral obligations to members of different groups. These three issues are fundamental (definitions, acquisition, and age-related change), but they clearly do not exhaust all major points of debate about a complex construct such as morality. We hope that addressing these concerns will help integrate research on how morality develops during the first year of life.

## Research on Early Development Needs a Definition of Morality

We propose that an investigation of early moral development requires a definition of morality and other key concepts. In our view, explicit definitions of key terms are crucial to the accumulation of knowledge (Dahl, 2014; Dahl and Killen, 2018). In contrast, some scholars have explicitly stated that morality does not need to be defined and that the inquiry of moral concepts necessitates asking participants what morality means to them, noting that the word “morality” is used in a variety of ways (Greene, 2007; Haidt and Graham, 2007; Wynn and Bloom, 2014). We argue that morality, perhaps even more than other concepts, requires definition and criteria. One problem with defining morality in terms of what people label as moral is that morality can become relativistic; whatever action or belief any one person, group, or culture deems to be “moral” is so (for discussions, see Kohlberg, 1969; Turiel, 2002, 2015a). Moreover, when researchers do not define morality, it is difficult to determine whether disagreements among scholars result from different uses of the word “moral” or from different empirical claims. Indeed, explicit definitions of phenomena for investigation reflect the core of scientific analysis and are crucial for empirical evaluation of scientific claims.

In our work, we have defined morality as prescriptive norms concerning others’ welfare, rights, fairness, and justice (Killen and Rutland, 2011; Turiel, 2015a; Dahl and Killen, 2018). The research task is to determine when children’s judgments reflect these criteria. This definition of morality stems from neo-Kantian philosophical accounts of morality (Turiel, 1983; Smetana et al., 2014). Within our framework, morality is not the only basis for evaluative judgments: children and adults also make judgments about conventional, religious, and personal safety considerations (see Killen and Smetana, 2015). The usefulness of defining morality in terms of others’ welfare, rights, fairness, and justice is now supported by a large body of research showing that children and adults distinguish moral considerations from considerations about social conventions, and from matters of personal choice (Killen and Smetana, 2015). For instance, most children across different communities think that it would be wrong to harm others even when parents or teachers condone it. In contrast, most children view conventional issues, such as dress codes or forms of address, as alterable by authorities. We are not asserting that there is only one definition of morality; our main point is that an explicit definition of morality is crucial for avoiding major miscommunication, and promoting accumulation of knowledge, in research on early moral development.

## Early Morality is Constructed, and is Neither Innate nor Learned

While psychological research in the first half of the 20^th^ century often framed one of the fundamental questions about psychological behavior as whether it was innate or learned, extensive research has subsequently undermined the dichotomy between innate and learned characteristics. In fact, all developmental transitions involve genetic, cellular, neural, behavioral, and environmental processes (Gottlieb, 1991; Spencer et al., 2009; Moore, 2015).

Children construct morality through reciprocal interactions with their environments (Dahl and Killen, 2018). The constructivist view does not seek to separate innate and learned elements of morality (Piaget, 1932). This view is also supported by evidence that children have an abundance of morally relevant experience from early in life, involving helping and being helped as well as harming and being harmed (Reddy et al., 2013; Dahl, 2015, 2016a,b; Hammond et al., 2017). Through these experiences, children come to critically evaluate norms from parents and others (Dahl and Kim, 2014; Dahl, 2016b; Dahl and Killen, 2018).

The constructivist viewpoint differs from contemporary nativist and learning views of moral development. In discussions of innate characteristics, it is often unclear how to determine whether some characteristic is “innate” (Dahl, 2014; Turiel and Dahl, in press). It is biologically implausible that any characteristic would develop irrespective of environmental processes. Some have proposed that we infer characteristics to be innate whenever the characteristics develop in the absence of relevant experience (Bloom, 2012; Hamlin, 2013). However, for morality, virtually any social interaction is a relevant experience. From birth, most infants interact with people who help and comfort them, for instance by feeding them or responding to their crying (Richards and Bernal, 1972; Tronick, 1989; Hammond et al., 2017). An infant who develops in the absence of morally relevant experiences would not develop at all.

Importantly, the constructivist view also differs from learning or socialization views of moral development. Socialization and learning views portray moral development as a process of complying with the norms and views of one’s community (Kochanska and Aksan, 2006; Grusec et al., 2014), leading to a relativistic theory of morality. In contrast, the constructivist view proposes that children acquire generalizable obligations about the fair and equal treatment of others through an active process, one that involves abstracting, interpreting, and evaluating social experiences, sometimes agreeing and sometimes challenging the norms held by one’s community (Nucci, 2005). Children also construct other evaluative concepts through social experiences, for instance by learning about social conventions or religious norms adopted by their parents, and other community members (Turiel, 1983; Killen and Smetana, 2015).

In proposing a constructivist approach, we seek to reorient research on early moral development. Rather than asking *whether* a given capability is innate or due to experiential factors, research can investigate *how* children construct morality through reciprocal interactions.

## Studying Developmental Change

Developmental research is the study of change. Yet, recent discussions of early moral development have often emphasized continuities concerning the presence of moral knowledge between infants and adults. Some researchers have proposed that infants make moral judgments, and possess altruistic motives, around the first birthday (Warneken and Tomasello, 2006; Bloom, 2013; Hamlin, 2013; Wynn and Bloom, 2014; Warneken, 2016). Contrasting with this emphasis on continuity, researchers have recommended greater attention to developmental change in moral development (Kagan, 2008; Killen et al., 2015; Dahl and Freda, 2017; Sommerville, 2018). These age-related changes include conceptual advancements, coordination of knowledge, and priority of certain moral principles over others. These gradual changes reflect new understandings about morality that were not present at younger ages. Here, we call for greater attention to developmental change in research on early moral development through discussions of helping behavior and research on children’s judgments of group-based social inequalities.

### Developmental Changes in Orientations Toward Helping

How do judgments of helpful actions develop? We assert that helping behavior, alone, is not necessarily “moral” behavior but reflects a first step toward the acquisition of morality. In some contexts, individuals judge helping as morally good or even obligatory, such as when it involves helping others from harm. In other situations, however, helping is viewed as undesirable and morally repugnant, such as helping someone cheat or steal (Miller et al., 1990; Kahn, 1992; Killen and Turiel, 1998; Turiel, 2015b). Thus, evaluations of helping behavior incorporate the goal of the action, and the basis for the motivation to help another person.

Early in life, children have experiences with helping and being helped by others. Most infants help others around the first birthday (Warneken and Tomasello, 2007; Sommerville et al., 2013; Dahl, 2015; Hammond et al., 2017). In one common laboratory paradigm, an adult accidentally drops a pen or a paperclip and unsuccessfully reaches for it. Infants commonly hand back the dropped object to the experimenter (Warneken and Tomasello, 2006; Warneken, 2013). In everyday life, 1-year-olds participate in a variety of chores, including putting toys away, laundry, self-care, and cleaning (Rheingold, 1982; Dahl, 2015; Hammond et al., 2017).

We propose that infants’ earliest helping behaviors are based on a desire to participate in social interactions, and are not accompanied by moral judgments that helping is good or required (Dahl and Paulus, in press; Miller et al., 1990; Kahn, 1992; Killen and Turiel, 1998; Turiel, 2015b). First, infants are not very reliable helpers. Infants who help on one trial do not always help on another, and often opt to play instead of helping (Warneken and Tomasello, 2006; Waugh and Brownell, 2017). Infants’ unreliable helping is striking because, in these studies, infants could help at minimal cost (Rheingold, 1982; Warneken et al., 2007). Second, when infants begin to help, they do not appear broadly concerned with others’ welfare. While infants on average become more helpful early in the second year of life, they also use more interpersonal force in this period, sometimes hitting or kicking others for no apparent reason and without visible signs of anger or distress (Hay, 2005; Dahl, 2015, 2016a).

Finally, infants do not make categorical judgments based on moral concerns (Dahl, 2014; Dahl and Freda, 2017). Although infants and toddlers prefer to reach and look toward helpful puppets over hindering puppets, they also show such preferences based on non-moral characteristics such as food preferences (Hamlin et al., 2013; Wynn, 2016). Moreover, infants’ preferences are relative, not qualitative: These studies show that infants prefer one puppet over another, but do not show that infants view some puppets as bad or wrong (Vaish et al., 2010; Dahl et al., 2013).

Infants’ desire to participate in chores and other adult activities is an important developmental precursor to morality. Still, this desire does not constitute a moral concern. Orientations toward helping undergo transformations between infancy and later childhood (Dahl et al., 2018). By 3–4 years of age, children make categorical judgments about right and wrong based on concerns with welfare and rights (Nucci and Weber, 1995; Smetana et al., 1999; Schmidt et al., 2012; Dahl and Kim, 2014; Killen and Smetana, 2015; Josephs and Rakoczy, 2016). Hence, preschoolers have developed obligatory concepts and concerns regarding others’ welfare and apply these in social situations. Past research indicates that children make judgments of right and wrong about helping by age 8, and likely before (Kahn, 1992; Nucci et al., 2017; Van de Vondervoort and Hamlin, 2017). More research is needed to explain the development of moral orientations toward helping, from a desire for participation to judgments based on concerns with welfare and rights (Dahl and Paulus, in press).

### Developmental Changes in Intergroup Attitudes and Moral Judgments

As children grow older, they also encounter acts that involve members of other groups. Over the past decade, research in developmental psychology has examined the origins of morality in concert with the emergence of social equality, or how young children apply their moral judgments to intergroup contexts (Schmidt et al., 2012; Hetherington et al., 2014; Weller and Lagattuta, 2014; Killen et al., 2015). Do young children distribute resources by giving more to their ingroup than to an outgroup when both groups are equally meritorious? Do moral judgments play a positive force, enabling children to reject peers who promote stereotypic or prejudicial attitudes (Killen et al., in press; Mulvey, 2016; Rutland and Killen, 2017). These are fundamental questions regarding how morality, defined as the fair and equal treatment of others, is applied in situations in which group identity is salient (Nesdale, 2004).

Group affiliation is necessary for human survival (Tomasello, 2016). At the same time, many forms of group loyalty are unfair, resulting in negative treatment toward others, and particularly those perceived as members of outgroups. Children and adults in many cultures view group norms related to societal conventions as contextually bound and consensus-driven whereas moral principles are generalizable and obligatory (Smetana et al., 2014), reflecting *continuity* in thinking about group norms. As early as 3–6 years of age children, view moral norms as obligatory, and view group loyalty as relative to the type of loyalty required, such as whether the loyalty is conventional (wearing the team colors) or moral (Liberman et al., 2018; Rizzo et al., 2018).

What changes with age is the recognition of the obligation and orientation to reject unfair group norms, which requires taking a number of contextual factors into account (Mulvey, 2016). A series of age-related shifts has been documented during early childhood in which children begin to actively challenge unfair group norms and view exclusion from groups based on stereotypic expectations of individuals as wrong (see Killen et al., in press). One finding that stands out is that, with age, knowledge about groups is related to children’s increased ability to rectify inequalities (Elenbaas and Killen, 2016a). Further, an increase in psychological knowledge about others’ intentions (such as theory of mind) enables children to reject exclusion as well as the denial of resource allocations based on stereotypic norms (Mulvey et al., 2016b; Rizzo and Killen, 2018).

Whereas 5 to 6-year-olds distribute resources equitably when faced with two characters, one who has lots of resources (e.g., wealthy) and one who does not (e.g., poor), 3 to 4-year-olds allocate equally (even though they recognize that equity would be legitimate if another child gave more to those who have less) (Rizzo and Killen, 2016). When asked about whether others would reduce inequalities, 5 to 6-year-olds, but not 3 to 4-year-olds expect individuals to seek more for their ingroup if they are told that the group prefers their ingroup. Younger children do not take information about ingroup bias into account when asked what groups will do (Elenbaas and Killen, 2016b).

With increasing theory of mind abilities, 4 to 6-year-old children allocate resources based on merit in gender non-stereotypic contexts in contrast to children without theory of mind who fail to reward meritorious behavior when the activity does not conform to the gender stereotype (e.g., boys making dolls or girls making trucks) (Mulvey et al., 2016a; Rizzo and Killen, 2018). Moreover, children who pass false belief theory of mind are more likely than children who fail to expect others to challenge gender stereotypes about what toy to play with and were also more supportive of those challenges (Mulvey et al., 2016b). Further, with age (from 5–6 years to 10–11 years) knowledge about group inequalities based on race has been shown to be related to decisions to rectify inequalities when distributing resources, with younger children less aware and more likely to perpetuate the inequality than older children (Elenbaas and Killen, 2016b). Thus, the emergence of morality reflects age-related changes regarding incorporating information about group identity and group norms into moral decisions and judgments.

## Conclusion and Future Directions

This article proposes a constructivist approach to early moral development. We made three main points. First, a definition of morality is key to studying morality: definitions guide empirical research questions and hypotheses. Second, transitions in early moral development involve genetic, environmental, and social-cognitive factors. Morality and its precursors cannot be split into some characteristics that are innate and others that are learned. Third, an account of the origins of morality requires investigations of the processes that lead to the acquisition of new forms of moral judgments, reasoning, and concerns. In the area of helping, research that connects early helping behavior with evaluative judgments about helping in childhood would be fruitful. To extend research on morality in intergroup contexts, documenting the factors that enable children to challenge inequalities and unfair treatment would be impactful. We believe that scholars would benefit from providing explicit definitions of key terms, abandoning the dichotomy between innate and learned characteristics, and considering developmental change in research on early morality and its precursors.

## Author Contributions

Both authors contributed equally to the conceptualization and writing of this article.

## Conflict of Interest Statement

The authors declare that the research was conducted in the absence of any commercial or financial relationships that could be construed as a potential conflict of interest.
